# Quality of life in adults with Gilles de la Tourette Syndrome

**DOI:** 10.1186/1471-244X-12-109

**Published:** 2012-08-13

**Authors:** Isabelle Jalenques, Fabienne Galland, Laurent Malet, Dominique Morand, Guillaume Legrand, Candy Auclair, Andreas Hartmann, Philippe Derost, Franck Durif

**Affiliations:** 1 CHU Clermont-Ferrand, Pôle de Psychiatrie, Service de Psychiatrie de l’adulte et psychologie médicale A, Hôpital Gabriel Montpied, Clermont-Ferrand, F-63003, France; 2 CHU Clermont-Ferrand, Pôle de Psychiatrie, Service de Psychiatrie de l’adulte B, Hôpital Gabriel Montpied, Clermont-Ferrand, F-63003, France; 3 CHU Clermont-Ferrand, Délégation à la Recherche Clinique et à l’Innovation, Hôpital Gabriel Montpied, Clermont-Ferrand, F-63003, France; 4 CHU Clermont-Ferrand, Santé Publique, Clermont-Ferrand, France; 5 Département de Neurologie, Pôle des Maladies du Système Nerveux, Groupe Hospitalier La Pitié-Salpêtrière, Centre de référence ‘Syndrome Gilles de la Tourette, Paris, F-75013, France; 6 CHU Clermont-Ferrand, Neurologie, Hôpital Gabriel Montpied, Clermont-Ferrand, F-63003, France; 7 Clermont Université, Université Clermont 1, UFR Médecine, EA 3845, Clermont-Ferrand, F-63001, France

**Keywords:** Gilles de la Tourette Syndrome, Quality of life, Physical and psychological health status, Psychology, Psychiatric disorders, Depression

## Abstract

**Background:**

Few studies have used standardized QOL instruments to assess the quality of life (QOL) in Gilles de la Tourette Syndrome (GTS) patients. This work investigates the QOL of adult GTS patients and examines the relationships between physical and psychological variables and QOL.

**Methods:**

Epidemiological investigation by anonymous national postal survey of QOL of patients of the French Association of Gilles de la Tourette Syndrome (AFGTS) aged 16 years or older. The clinical and QOL measures were collected by four questionnaires: a sociodemographic and GTS-related symptoms questionnaire, the World Health Organization Quality Of Life questionnaire (WHOQOL-26), the Functional Status Questionnaire (FSQ), and a self-rating questionnaire on psychiatric symptoms (SCL-90), all validated in French. We used stepwise regression analysis to explicitly investigate the relationships between physical and psychological variables and QOL domains in GTS.

**Results:**

Questionnaires were posted to 303 patients, of whom 167 (55%) completed and returned them. Our results, adjusted for age and gender, show that patients with GTS have a worse QOL than the general healthy population. In particular, the “Depression” psychological variable was a significant predictor of impairment in all WHOQOL-26 domains, psychological but also physical and social.

**Conclusions:**

The present study demonstrates a strong relationship between QOL in GTS and psychiatric symptoms, in particular those of depression.

## Background

Gilles de la Tourette syndrome (GTS) is a developmental neuropsychiatric disorder characterized by multiple motor tics and one or more vocal tics occurring over a period of more than one year [1]. The age of onset of GTS ranges from 2 to 18 years, with a commonly reported mean of 7 years. Recent studies have suggested that GTS occurs in at least 1% of the worldwide population, with a male/female ratio of 3:1, and a prevalence between 0.4% and 3.8% for ages from 5 to 18 years [2]. Thus, the disease begins during childhood and develops in a succession of periods with variable aggravation or remission of the tics. Most patients show improvement at the end of adolescence, but symptoms can persist into adulthood in about one third of those affected [3]. It is now recognized that GTS is a complex pathology with a high occurrence of comorbid neuropsychiatric disorders (up to 90% of GTS patients) [2,4-7]. The most common are attention-deficit/hyperactivitiy disorder (ADHD) and obsessive-compulsive disorder (OCD) [8]. Depression, anxiety, self-injurious behaviours (SIB) and conduct disorders (CD) are also common in adult GTS subjects [7,9]. Major handicaps for patients affected by GTS are delayed diagnosis, non-specialist management, and inadequate educational and social support [8].

Recent studies have described the psychological, familial, social and educational impact of tics and associated psychopathologies on children with GTS [10-12]. In adult GTS patients only a few studies have used standardized QOL instruments for assessment. Importantly, the two major reports on the subject [13,14] were made in tertiary care centres whereas our study investigated the QOL of GTS patients and examined the relationship between physical and psychological variables and quality of life in a consecutive, unselected sample. Specifically, we conducted an anonymous national postal survey of QOL among members of the French Association of Gilles de la Tourette Syndrome (AFSGT) aged 16 years or older. Members completed and returned self-rating questionnaires that had been validated in French and thus could be relied on to correctly estimate health and social problems. We expected an impairment of all domains of QOL and a consistent relationship with the occurrence of comorbid psychiatric disorders.

## Methods

### Patients

A total of 303 patients aged 16 years or older of the AFSGT were identified and sent self-rating questionnaires in March 2007, with a second anonymous mailing in June 2007 [15]. Only fully completed questionnaires were used. Of the 303 patients, 125 completed and returned questionnaires after the first mailing, and 42 after the second. Thus 167 patients were included in this study. The study received approval from the inter-regional ethics committee of Rhône-Alpes-Auvergne in Grenoble. Patient information was recorded on the first page of the questionnaire. Participants gave informed consent by returning the questionnaire.

### Data collection procedures and instruments

The clinical and QOL measures were collected by four questionnaires: a sociodemographic and GTS-related symptoms questionnaire, the WHOQOL-26 (World Health Organization Quality Of Life) questionnaire, the FSQ (Functional Status Questionnaire), and the SCL-90 (self-rating questionnaire on psychiatric symptoms), all validated in French [16-22].

### Sociodemographic and GTS-related symptoms questionnaire

At baseline, socio-demographic data on gender, age, lifestyle, marital status, education, and employment status were collected. Clinical data related to motor and vocal tics, and their severity, age at onset, age at diagnosis and daily frequency. Management was identified by questions about treatment, specialist or generalist management, age at initiation of management, psychotherapy, and compliance with care. The patient’s own subjective view about the severity of tics and of GTS was collected by visual analogue scales (VAS): range no tic to maximum severity of tics; no GTS to maximum severity of GTS. When responding to a VAS item, respondents specified their level of agreement with a statement by indicating a position along a continuous line between two end-points. The sensitivity and reproducibility of the VAS results are broadly satisfactory [23].

### SCL-90 R (self-rating questionnaire about psychiatric symptoms)

The SCL-90 R is the latest version of a self-report inventory of symptomatic complaints developed from previous inventories [16]. It is a 90 item instrument which was chosen to measure 10 postulated factors: depression, somatisation, anxiety, phobic anxiety, obsessive-compulsive, interpersonal sensitivity, anger-hostility, psychoticism, paranoid ideation, and sleep and concentration difficulties. We used the French translation by Pariente and Guelfi [21]. Each of the 90 items corresponds to a sentence which the subject rates on a 5-point scale: “1: not at all” to “5: extremely” according to his or her mood state over the previous 7 days. The SCL-90 R manual gives a global score (from 90-450) and specific scores for each domain. It also provides three other indexes computed from the item scores: the General Symptomatic Index (GSI), which represents overall severity, the Positive Symptom Total (PST), which assesses the diversity of symptoms, and the Positive Symptom Distress Index (PSDI), which takes into account the patient’s own subjective view about the degree of discomfort for each symptom. Its validity is well documented [24-26].

### WHOQOL-26 (World Health Organization Quality Of Life)

The WHOQOL-Bref or WHOQOL-26 is an abbreviated version of the WHOQOL-100 quality of life assessment instrument, which is a generic, multidimensional measure for subjective assessment of QOL [22,27-29] The WHOQOL-26 was developed to allow a brief and accurate assessment of QOL in routine clinical work, large scale-epidemiological studies and clinical trials [22]. It comprises one question for each of the 24 facets in the WHOQOL-100 belonging to one of the following domains: physical health (7 items), psychological health (6 items), social relationships (3 items) and environment (8 items). Facets incorporated within the “physical” domain are: activities of daily living, dependence on medicinal substances and medical aids, energy and fatigue, mobility, pain and discomfort, sleep and rest, and work capacity. “Psychological” facets allow assessment of bodily image and appearance, negative and positive feelings, self-esteem, spirituality and personal beliefs, and capacity of learning, memory and concentration. Other facets allow detailed assessment of “social relationships”: personal relationship, social support, sexual activities; and of “environment”: financial resources, physical safety, accessibility and quality of health and social care, home environment, opportunities of acquiring new information and skills, participation in and opportunities for leisure activities, transport, and quality of environment (e.g. pollution, noise). Two further questions are global indicators of QOL and satisfaction with health. In total, it has 26 items, each with a five-point Likert scale. High scores indicate good QOL. Apart from the four domain- and two well-being scores an overall score is used including all items. The time of reference is the previous two weeks. In earlier research, analyses of the internal consistency, item-total correlations, discriminant validity, and construct validity through confirmatory factor analysis, indicated that the WHOQOL-26 had good to excellent reliability and validity in a population of psychiatric patients [22,30]. We used the French translation by Leplège [18].

### FSQ (Functional Status Questionnaire)

The Functional Status Questionnaire (FSQ) is a brief, standardized, self-administered questionnaire designed to provide a comprehensive and feasible assessment of physical, psychological, social and role function in ambulatory patients [17,19,20]. It comprises questions for each of the following sections: physical functions (basic Activities of Daily Living or ADL e.g. taking care of oneself, moving in or out a bed or chair, walking indoors; intermediate ADL e.g. running, home maintenance, shopping, driving), mental health (items ask patients how they viewed their personality, e.g. nervous/peaceful, downhearted/happy), work performance (quantitative or qualitative limitations because of impaired health), social activities (difficulty visiting with relatives or friends, participating in community activities, taking care of other people) and the quality of their social interactions (isolating oneself, acting affectionate toward others, being irritable toward those around, making unreasonable demands on family or friends). The FSQ can be completed and computer-scored in minutes to produce a one-page report which includes six summated-rating scale scores and six single-item scores. The clinician can use this report both to screen for and to monitor patients' functional status. In earlier research, findings demonstrated that the FSQ produces reliable sub-scales with construct validity [17]. We used the validated French translation by Martin [19,20].

Since the QOL assessment by postal survey included self-rating questionnaires, a pilot study was needed to assess their feasibility. We sent questionnaires to 21 patients attending the GTS specialized follow-up clinic of university hospitals in Clermont-Ferrand and Paris. Twenty questionnaires were returned fully completed. The remaining one was unusable owing to numerous invalid answers. Four others had one answer missing, which did not invalidate overall assessment. The mean time to complete the questionnaires was 31.5±10.5 minutes. Because the questionnaires were satisfactorily completed by subjects, only a few adjustments were made to make them easier to read and more attractive.

### Statistical analysis

Data were analysed using the Statistical Package for Social Sciences (SPSS, version 12.0 for Windows). All statistical tests used a two-sided risk α of 5%.

Means of each dimension of the WHOQOL-26 were compared between GTS patients (n = 167) and data extracted from the National Institute of Prevention and Health Education (NIPHE), using Student’s *t*-test. NIPHE is a nationally representative sample (n = 3560) obtained in national telephone surveys that cover the development of knowledge, opinions, attitudes and behaviours of the French population in the field of health [30]. Results were confirmed by multiple linear regression adjusted for age and gender.

Stepwise regression analyses adjusted for age and gender were performed to determine in the GTS sample the relationship between physical and psychological variables (VAS Tic, VAS GTS, SCL-90 R independent variables) and quality of life and functional status (domain scores dependent variables).

## Results

The sociodemographic characteristics of the 167 patients are presented in Table 1. The mean age was 29.3 years (SD 12.0, range 16-68). The mean age at onset of disease was 8.8 years (SD 4.3). The mean age at diagnosis of disease was 17.5 years (SD 9.4). Eighty nine per cent of patients (n = 148) reported tics at the time of the study. About 32% of the patients had no medication. The main drug treatments were atypical neuroleptics (54.5%), antidepressants (38.3%), anxiolytics (17.4%) and psychostimulants (3%); 47.3% had neurological specialist management, 31.1% psychiatric and 10.2% psychological management.

**Table 1:** Sociodemographic characteristics of patients with Gilles de la Tourette Syndrome (n = 167)

** Characteristics**	***n***	**%**
Gender		
Male	124	74.3
Female	43	25.7
Marital status/Accomodation		
Single	33	19.8
Partner/married	50	29.9
With parents	76	45.5
Institution	3	1.8
Other	4	2.4
Unknown	1	0.6
Education		
No exam	29	17.4
Graduate Certificate	32	19.2
Technical training	37	22.2
Bachelor	28	16.8
University graduate	40	24.0
Unknown	1	0.6
Employment		
Unemployed	32	19.2
At school, at university	48	28.7
Normal employment	11	6.6
Special employment	59	35.3
Housewife	5	3.0
Retired	4	2.4
Unknown	8	4.8

The mean score of severity of tics collected by VAS was 43.4 (SD 22.9); 141 of the 148 patients reporting tics at the time of the study completed the score.

Table 2 shows the SCL-90 R results. All factors were impaired. The most affected factors were the obsessive-compulsive factor, interpersonal sensitivity, depression and hostility.

**Table 2:** Psychiatric symptoms: mean scores in the different factors of the SCL-90R

** Factors**	***mean***	***SD***
Obsessive-compulsive	1.23	0.78
Interpersonal sensitivity	1.22	0.78
Depression	1.08	0.78
Hostility	1.04	0.80
Paranoid ideation	0.94	0.90
Anxiety	0.91	0.75
Sleep and concentration difficulties	0.85	0.71
Phobic anxiety	0.71	0.60
Somatisation	0.68	0.75
Psychoticism	0.59	0.67
		0.57

SD: standard deviation.

Most affected factors indicated by higher scores.

Results of the WHOQOL-26 of GTS patients compared to those of the healthy population (NIPHE sample) are shown in Table 3. In comparison to a healthy population, GTS patients had significantly lower scores in all domains indicating worse QOL: physical (*t*-Test, t = -5.57, p<0.0001), psychological (*t*-Test, t = -8.29, p<0.0001), social (*t*-Test, t = -7.7, p<0.0001) and environment (*t*-Test, t = 2.56, p<0.01).

**Table 3:** Comparison of WHOQOL-26 results of GTS patients with healthy population of NIPHE sample

**WHOQOL-26 Domains**	**GTS *Mean (SD)***	**NIPHE sample *Mean (SD)***	***Student’s test p value***
Physical health	13.77 (3.30)	15.21 (2.52)	*<0.0001*
Psychological health	12.18 (3.05)	14.15 (2.31)	*<0.0001*
Social relationships	12 .97 (3.93)	15.31 (2.51)	*<0.0001*
Environment	14.64 (2.69)	15.18 (2.36)	*<0.001*

SD: standard deviation.

WHOQOL-26: World Health Organisation Quality of Life 26.

NIPHE: National Institute of Prevention and Health Education.

Worse QOL indicated by lower scores.

The six FSQ mean summated-rating scale scores are given in Table 4. GTS patients mentioned good physical function (mean basic ADL 96.02, SD 10.17; mean intermediate ADL 86.86, SD 18.22). Mean summated-rating scale score for psychological functions was clearly impaired (mean 52.92, SD 19.36). The two mean summated-rating scale scores for work performance and social activities were not dramatically impaired (mean work performance 80.83, SD 18.65; mean social activities 83.47, SD 22.96) but patients reported poor quality of social interactions (mean 69.32, SD 15.22).

**Table 4:** Functional Status Questionnaire (FSQ) mean GTS patients summated-rating scale scores

**FSQ Sections**	**GTS *Mean (SD)***	***Qualitative data***
Physical functions:		
Basic ADL	96.02 (10.17)	Good
Intermediate ADL	86.86 (18.22)	Good
Mental health	52.92 (19.36)	Clearly pathological
Work performance	80.83 (18.65)	Warning zone
Social activities	83.47 (22.96)	Warning zone
Quality of interactions	69.32 (15.22)	Pathological

SD: standard deviation; ADL: Activities of Daily Living; FSQ: Functional Status Questionnaire.

Summated-rating scale scores for basic ADL: “Good” 88-100, “Warning zone” 0-87; for intermediate ADL: “Good” 78-100, “Warning zone” 0-77;for mental health: “Good” 71-100, “Warning zone” 0-70; for work performance: “Good” 79-100, “Warning zone” 0-78; for social activities: “Good” 79-100, “Warning zone” 0-78; for quality of interactions: “Good” 70-100, “Warning zone” 0-69.

Table 5 gives figures about the influence of the severity of tics and GTS disease (VAS) and psychiatric symptoms (SCL90R) upon each dimension of the WHOQOL 26, when adjusted for age and gender. The variable “depression” was significantly associated with all WHOQOL-26 domains (Physical health domain: p<0.0001; Psychological health domain: .p<0.0001; Social relationships domain: p<0.0001; Environment domain: p<0.0001).Figures about the influence of the severity of tics and GTS disease (VAS) and Psychiatric Symptoms (SCL90R) upon each dimension of the FSQ, with results adjusted for age and gender, are given in Table 6. The variable “Depression” was significantly associated with the domains of “mental health” (p<0.0001) and “quality of interactions” (p<0.0001) FSQ.

**Table 5:** Influence of the severity of tics, GTS and psychiatric symptoms on each dimension of QOL

**WHOQOL-26 domains**	**Independent variables**	**R2 total**	***p value***
Physical health	Depression*	0.4925	*<0.0001*
	**VAS GTS**	0.6003	*0.0027*
	Somatisation*	0.6120	*0.0478*
Psychological health	Depression*	0.5447	*<0.0001*
Social relationships	Depression*	0.2973	*<0.0001*
	Somatisation*	0.3669	*0.0002*
	Sensitivity*	0.4092	*0.0023*
	Anxiety*	0.4342	*0.0161*
	**VAS GTS**	0.4640	*0.0074*
Environment	Depression*	0.2332	*<0.0001*
	**VAS GTS**	0.2929	*0.0009*
	Somatisation*	0.3355	*0.0039*

VAS GTS visual analogue mean scores severity of Gilles de la Tourette Syndrome Psychiatric symptoms: SCL-90R: Symptom Checklist 90 Revised

WHOQOL-26: World Health Organisation Quality of Life-26; stepwise regression analysis.

* One of 10 factors of SCL-90R.

**Table 6:** Influence of the severity of tics, GTS and psychiatric symptoms on dimensions of the FSQ

**FSQ Domains**	**Independent variables**	**R2 total**	***p value***
Physical - basic ADL	Psychotic*	0.0961	*0.0002*
Physical-intermediate ADL	Phobic anxiety*	0.2269	*<0.0001*
	**VAS GTS**	0.3301	*<0.0001*
	Somatisation*	0.3544	*0.0254*
Mental health	Depression*	0.5925	*<0.0001*
	**VAS GTS**	0.6154	*0.0050*
Work performance	Obsessive-compulsive*	0.2248	*<0.0001*
	**VAS TIC**	0.2674	*0.0432*
Social activities	Phobic anxiety*	0.2272	*<0.0001*
	**VAS TIC**		
	Sensitivity*	0.2788	*0.0022*
		0.3191	*0.0054*
Quality of social interactions	Depression*	0.2888	*<0.0001*
	Hostility*		
	Obsessive-compulsive*	0.3427	*0.0010*
		0.3640	*0.0345*

FSQ: Functional Status Questionnaire.

ADL: Activities of Daily Living.

VAS GTS visual analogue mean scores severity of Gilles de la Tourette Syndrome.

VAS TIC: visual analogue mean scores of severity of Tics.

SCL-90R: Symptom Checklist 90 Revised of psychiatric symptoms.

Stepwise regression analysis.

*One of 10 factors of SCL-90R.

We examined whether there was an interaction between tic severity and depression SCL-90R domain score in predicting QOL. Hierarchical linear regressions were computed, with QOL and functional status domain scores as independent criteria, and adjusted for age. In step 1, the tic severity and depression SCL-90R score were entered; in step 2 their interaction was entered. There was no significant effect of the interaction between tic severity and depression for all QOL domains. Similar non-significant results were found with FSQ dimensions (Tables 7 and 8).

**Table 7:** Hierarchical linear regression analyses adjusted for age predicting WHOQOL-26 domain scores from SCL-90R depression score

**Order of entry**	**β**	**Significance of β**	**R**^**2**^	**ΔR**^**2**^	**ΔF**	**Significance of ΔF**
		***(p-value)***				***(p-value)***
**Physical health**						
Step 1			0.5428	0.5428	53.43	*<0.0001*
VAS TIC	-0.30	*0.0053*				
SCL-90R depression score	-0.71	*<0.0001*				
Step 2			0.5444	0.0016	0.48	*0.4898*
Interaction of VAS TIC and SCL-90R depression score	0.13	*0.4898*				
**Psychological health**						
Step 1			0.5504	0.5504	55.10	*<0.0001*
VAS TIC	-0.12	*0.2590*				
SCL-90R depression score	-0.89	*<0.0001*				
Step 2			0.5420	0.0048	1.45	*0.2303*
Interaction of VAS TIC and SCL-90R depression score	0.22	*0.2303*				
**Social relationships**						
Step 1			0.2926	0.2926	18.75	*<0.0001*
VAS TIC	-0.06	*0.6402*				
SCL-90R depression score	-0.58	*0.0010*				
Step 2			0.2931	0.0005	0.10	*0.7509*
Interaction of VAS TIC and SCL-90R depression score	0.07	*0.7509*				
**Environment**						
Step 1			0.2533	0.2533	15.38	*<0.0001*
VAS TIC	-0.24	*0.0792*				
SCL-90R depression score	-0.60	*0.0008*				
Step 2			0.2599	0.0065	1.19	*0.2769*
Interaction of VAS TIC and SCL-90R depression score	0.25	*0.2769*				

VAS TIC: visual analogue mean scores of severity of Tics; SCL-90R: Symptom Checklist 90 Revised of psychiatric symptoms.

**Table 8:** Hierarchical regression analyses adjusted for age predicting FSQ dimension scores from SCL-90R depression score

**Order of entry**	**β**	**Significance of β *(p-value)***	**R**^**2**^	**ΔR**^**2**^	**ΔF**	**Significance of ΔF *(p-value)***
**Physical – basic ADL**						
Step 1			0.0770	0.0770	3.81	*0.0117*
VAS TIC	-0.09	*0.5587*				
SCL-90R depression score	-0.07	*0.7152*				
Step 2			0.0789	0.0019	0.28	*0.5961*
Interaction of VAS TIC and SCL-90R depression score	-0.14	*0.5961*				
**Physical – intermediate ADL**						
Step 1			0.2977	0.2977	19.36	*<0.0001*
VAS TIC	-0.16	*0.2185*				
SCL-90R depression score	-0.18	*0.2872*				
Step 2			0.3049	0.0072	1.41	*0.2372*
Interaction of VAS TIC and SCL-90R depression score	-0.26	*0.2372*				
**Mental health**						
Step 1			0.5972	0.5972	67.70	*<0.0001*
VAS TIC	-0.01	*0.9567*				
SCL-90R depression score	-0.69	*<0.0001*				
Step 2			0.5980	0.0008	0.28	*0.6001*
Interaction of VAS TIC and SCL-90R depression score	-0.09	*0.6001*				
**Work performance**						
Step 1			0.1489	0.1489	4.20	*0.0085*
VAS TIC	0.14	*0.5002*				
SCL-90R depression score	0.17	*0.4910*				
Step 2			0.1976	0.0487	4.31	*0.0514*
Interaction of VAS TIC and SCL-90R depression score	-0.58	*0.0514*				
**Social activities**						
Step 1			0.2649	0.2649	16.34	*<0.0001*
VAS TIC	-0.07	*0.5866*				
SCL-90R depression score	-0.11	*0.5390*				
Step 2			0.2775	0.0126	2.36	*0.1271*
Interaction of VAS TIC and SCL-90R depression score	-0.35	*0.1271*				
**Quality of social interactions**						
Step 1			0.2936	0.2936	18.98	*<0.0001*
VAS TIC	-0.02	*0.8817*				
SCL-90R depression score	-0.53	*0.0025*				
Step 2						
Interaction of VAS TIC and SCL-90R depression score	-0.01		0.2936	0.0000	0.00	*0.9486*
		*0.9486*				

VAS TIC: visual analogue mean scores of severity of Tics; SCL-90R: Symptom Checklist 90 Revised of psychiatric symptoms.

## Discussion

Our survey shows that GTS patients had a worse QOL than the general healthy population. These results are in agreement with those of two other studies performed on QOL in adults GTS. The first [13] measured QOL in 103 patients with a generic self-rating questionnaire SF-36 (Medical Outcome Study 36-Items Short-Form Health Survey), and a generic hetero-questionnaire QOLAS (Quality of Life Assessment Schedule). The authors concluded that patients with GTS had significantly worse QOL than the general population in all subscales of the SF-36 except the “vitality” subscale, but had better QOL than 145 patients with intractable epilepsy. However, QOL in GTS patients could be overestimated. The QOLAS was initially developed to assess patients suffering from epilepsy or dementia and so may underestimate health problems in a population of GTS patients. Secondly, the social dimension of the SF-36 is limited. SF-36 allows assessment of the limitations in functioning with a bias towards basic activities of everyday life and work activities, but it does not capture restrictions on activities due to the social stigma of GTS and feelings of embarrassment. In the second study, of Müller-Vahl et al. [14], which included 200 patients, the social dimension was not assessed. The quality of life scale used, the EQ-5D [31], is a very simple tool allowing for a very general assessment of quality of life and possesses only one item per dimension. It is mostly used in medico-economic studies [32]. Lastly, Müller-Vahl et al. [14] did not assess obsessive-compulsive symptoms whereas this dimension is represented in the SCL90R, as is depression, which Müller-Vahl et al. assessed using the Beck Depression Inventory (BDI) [14].

Studies of GTS in children and adolescents provide evidence of relationships between QOL and psychiatric comorbidity. In the study of Storch et al. 2007, for example, QOL as assessed by children and their parents seems to be influenced by the presence of psychiatric symptoms [10]. With relation to depressive symptoms in children, Eddy et al. 2010 reported that GTS patients had more depressive symptoms than control subjects and controls with epilepsy. However, results from these studies in children have yet to show a causal relationship between depression, tic severity and QOL [33].

We chose the WHOQOL-26 because it has good to excellent reliability and validity in neuropsychiatric patients [22]. It is easily administered and does not impose a great burden on the respondent. Furthermore, it is a sound cross-culturally valid assessment of QOL [22,27-29]. As social functioning is a key dimension in assessing QOL in GTS patients, we used the FSQ, which allows a distinction between good activities of everyday life, impaired social activities and work performance, and patients’ subjective views about poor quality of social interactions [17,19,20]. Using these different scales, which are reliable in estimating health and social problems in a patient group with a young mean age, we were able to show a dramatic impact of GTS in all domains (physical, psychological, social) of QOL and point up the need for a multidisciplinary approach to GTS. Recently, Cavanna et al [34] constructed and validated a new disease-specific patient-reported scale for the measurement of QOL in patients with GTS, the GTS-QOL. Response data analysis and item reduction methods led to a final 27-items with four subscales (psychological, physical, obsessional and cognitive) which take into account the complexity of the clinical picture of GTS. As a result, the GTS-QOL contains only three items covering questions of reverberations on social integration and support, and one item covering difficulties in ADL. Physical and cognitive factors contain many items covering specific symptoms of GTS (movement dyscontrol, phonic tics, repeating words and actions, copying people, unpleasant thoughts). The cognitive domain has four items (covering memory, losing items, difficulty finishing tasks and concentration) which are not contained in the generic QOL scales used in our study. The GTS-QOL has not yet been validated in French and could not be used in our study as it was published after the clinical data collection phase.

We sought to determine the relationship between QOL or functional status and severity of tics and GTS, and psychiatric comorbidities using a stepwise regression model adjusted for age and gender. In our study, the “depression” psychological variable is the only one significantly associated with all QOL domains of the WHOQOL-26, as in the findings of Müller-Vahl et al. [14] who, using multivariate analysis, reported that depression assessed by the BDI was the only variable significantly associated with reduced quality of life. The study of Elstner et al. [13] also reported an association between depression or anxiety and impaired QOL in adult GTS patients, although other factors contributed to QOL. However, these correlations were not calculated by multivariate analysis. Two investigations of young patients with GTS reported that generic Health Related Quality Of Life (HR-QOL) was primarily predicted by the comorbidities of OCD and ADHD rather than by tic severity [11,35]. The study of Cavanna et al. [34] confirms the relationships between QOL and physical and psychiatric variables using disease-specific patient-reported scales and standardized evaluations: GTS-QOL standardized mean scores were mainly affected by psychological and cognitive problems, followed by physical/activities of daily living and obsessive-compulsive themes.

We explored mediational relationships between depression, tic severity and QOL by hierarchical linear regression analysis. There was no significant effect of the interaction between depression and tic severity on the QOL domain scores.

It is noteworthy that depression and not tic severity was the main predictor of reduced QOL in both our study and that of Müller-Vahl et al. This should also suggest that tic severity is not directly related to mood, an observation that is in accordance with empirical clinical practice and deserves further exploration. A parallel can be drawn with Parkinson’s disease (PD), in which several studies have shown that reduced quality of life is mainly a function of depression and not motor status [36]. In contrast to GTS, however, depression in PD patients is not considered purely reactive but has an intrinsic organic nature due to the degeneration of noradrenergic and serotoninergic brainstem neurons. One might therefore speculate if increased depression in GTS is merely a reflection of psychosocial impairment in the broadest sense or whether there might be an organic susceptibility for depression and anxiety. The sex ratio observed in our sample (3:1) corresponds to that of other patient series [2,34]. We found, like other authors [13], that there was a high unemployment rate (19.2%) and poor academic achievement among patients (17.4% left school without qualifications). As in other studies, many patients lived with their family. Our sample was slightly younger than other populations included in previous studies, with the exception of that of Elstner et al. (29.3 years SD 12 in our study, 30.7 years SD 9.7 in Cavanna et al. [34], 28.7 years SD 8.6 in Elstner et al. [13,14], 34.9 years SD 11.8 Müller-Vahl et al. [14]). This can be partly explained by the choice of our sample: most members of the AFGST are children (not included in our study) and young subjects living with their families. Our patients are characteristic in that they are members of an association representing all stages of the disease and not the most severe cases attending GTS specialized follow-up clinics of university hospitals. We believe, therefore, that our sample is more representative of the general GTS population.

The results of the present study are in accordance with those of previous studies but several limitations should be considered when reviewing these findings. Our data were collected by an anonymous national postal survey of individuals who belonged to a patient association and we therefore did not know if all the patients fulfilled DSM-IV-TR criteria [1] for the diagnosis of GTS and for various GTS-associated psychiatric disorders. We questioned patients about their age at the time of diagnosis. Most patients nevertheless had specialist management (neurological and/or psychiatric and/or psychological) and we assessed psychological and psychiatric complaints by a validated self-report inventory, the SCL-90 R. Although we used VAS for assessing tic severity, which is a reproducible subjective scale sensitive to change in the assessment of symptoms, severity was not assessed by standardized and structured instruments such as the Yale Global Tic Severity Scale (YTGSS).

## Conclusion

The present study demonstrates a strong relationship between QOL in adult GTS patients and psychiatric symptoms, especially depression. This is important given that the main psychiatric comorbidity in GTS in terms of frequency is OCD but, with regard to QOL, we need to pay more attention to depression. We therefore propose that future studies perform a precise analysis of the relationships between GTS and depression, as has been previously suggested for ADHD and OCD [31]. It is also important to advocate a multidisciplinary approach, including the involvement of psychiatrists, to assess GTS patients with depression and other psychiatric comorbidities. A powerful treatment for depression, concurrent with tic treatment, may improve patient functioning and QOL more effectively.

## Competing interests

The authors declare that they have no conflict of interest.

## Author’s contribution

IJ conceived and designed the study, participated in obtaining funding and interpretation of data and wrote the paper. FG participated in the design of the study, data collection, drafting of the manuscript and obtaining funding. LM executed the statistical analysis and participated in interpretation of data. DM participated in data collection and assisted with the statistical analysis. GL and AH revised the manuscript critically for important intellectual content. Candy Auclair participated in the statistical analysis and interpretation of data. PD participated in the design of the study and drafting of the manuscript. FD participated in obtaining funding, in interpretation of data and revised the manuscript critically for important intellectual content. All authors read and approved the final manuscript.

## Funding

The authors thank the university hospital Clermont-Ferrand and the French Association of Gilles de la Tourette Syndrome (AFSGT) for their financial support.

## Pre-publication history

The pre-publication history for this paper can be accessed here:

http://www.biomedcentral.com/1471-244X/12/109/prepub
